# “I am No Longer Active, but I will Always Be a Crip”: A Longitudinal Ethnography (N = 1) of Gang Desistance in the Netherlands

**DOI:** 10.1007/s10612-025-09848-x

**Published:** 2025-09-12

**Authors:** Robert A. Roks

**Affiliations:** Erasmus School of Law, Erasmus University Rotterdam, Burgemeester Oudlaan 50, 3000 DR Rotterdam, The Netherlands

## Abstract

This article centers on the process of (gang) desistance of Jermaine, a man with a substantial criminal record who has been a member of the Dutch Crips gang for over a decade. Drawing on longitudinal ethnographic research, Jermaine’s process of desistance is outlined. This life story illustrates how much desistance should be understood as a struggle, accompanied by periods of relapsing into old patterns of (criminal) behavior. In addition to these more practical challenges in terms of act-desistance (Nugent and Schinkel, 2016), Jermaine's story showcases the importance of identity in the process of desistance. Above all, Jermaine's story indicates that for some, desistance means that the old criminal or gang identity does not need to be cast aside, but that it becomes part of the new identity of the desister.

## Introduction

On May 1, 2023, Jermaine^1^ and I stroll the streets of a small neighborhood in the Dutch city of The Hague that is known by members of the Dutch Crips gang as the “h200d” (Roks 2019). It is a familiar environment for the both of us; Jermaine has been hanging around the h200d since the late 1990s, and for me it has been my main fieldwork site since 2007. Moreover, it is the spot where we first met back in 2008. A lot has changed since we first met. Jermaine’s life has transformed from a respected gang member with a substantial criminal record to someone who uses his street and gang life experiences to mentor at-risk youth in educational and healthcare institutions. This is also the reason for our revisit to the neighborhood: Jermaine has been asked to talk about his criminal past for a short documentary and to explain how he tries to motivate young people to, in his words, *“leave the street game”*.

Criminology, Laub and Sampson (2001) write, has shown a preoccupation with answering the question why individuals start offending. How and why people stop committing crime, referred to by scholars as desistance—an *“ugly, difficult word”* according to Maruna (2025: 12)—, is less understood, something that also holds true for gang desistance. In part, this is because (gang) desistance is often approached as an event, as a demarcated point in time, rather than as a process. Moreover, most research on both desistance and gang desistance is *“synchronic rather than diachronic in nature”* (Rodgers 2023: 680). Studies, for instance, have relied on people looking back on their desistance experiences, something that is central to the seminal works by Laub and Sampson (2003) and Maruna (2001). Similarly, most of the available research on gang desistance and disengagement draws on *“indirect ethnographic observations, secondhand accounts, or reviews of the broader literature”* (Pyrooz and Decker 2011: 417), and *“only a handful of studies have information regarding their subjects either before or after gang involvement”* (Melde and Esbensen 2011: 515).

Given that previous studies have maintained a certain temporal and methodological distance, scholars of both desistance (Healy 2010; King 2013; Weijers 2022: 670–671) and gang desistance (Rodgers 2023) have opted for research that follow people during the process of desistance. To address this gap in the scholarly attention for (gang) desistance, this article draws on longitudinal ethnographic data on gang desistance. Although it was not my original intention to study desistance, it emerged as a central theme—specifically in the life of Jermaine—over the course of ethnographic fieldwork conducted among members of the Dutch Crips (Roks 2019). During my fieldwork between 2011 and 2014 for my PhD, Jermaine was one of my main research participants, and I noted that from mid-2012 onward, he began to disengage from gang and street life. In 2013, we met weekly and communicated daily via WhatsApp. It was during this period that I was able to closely document Jermaine’s process of desistance. For most of 2014, we remained in weekly contact—either in person, by phone, or via WhatsApp—and following the conclusion of my fieldwork, as of 2015, we spoke on average several times a month. For this paper, I will focus on the data I collected on Jermaine’s process of desistance between 2008 and 2015, albeit informed by more recent data collected in 2023.^2^

Inspired by the work of Maruna (2001) and Halsey et al. (2017), I adopt a phenomenological perspective on desistance, meaning I will foreground Jermaine’s experiences and meaning-making processes. In doing so, this paper offers a detailed account of the complexities when it comes to *“the maintenance of crime-free behavior in the face of life's obstacles and frustrations”* (Maruna 2001: 26). In outlining Jermaine’s process of desistance, I will pay attention to two spheres of desistance (Nugent and Schinkel 2016). First, I will examine what is referred to as “*act-desistance*”, or no longer engaging in criminality. In documenting this process, I will draw on what Contreras (2013: 210–211) has described as the *“wrong cultural capital”* or what is also known as “*street capital*” (Sandberg 2008; Sandberg and Pedersen 2011), i.e. knowledge and skills that are of value on the street, including the willingness and ability to use violence and know-how regarding illegal goods and services, as well as *“the resources available to individuals through social networks which allow them to thrive within the street field”* (Ilan 2013: 18). Outside of the confines of gang and street life, these Bourdieuian notions of capital (1985) have less value and can even be counterproductive to the process of desistance.

Second, I will shed light on Jermaine’s process of “*identity-desistance*”. This concept is used to refer to the internalization of a non-criminal or prosocial self-image (Giordano et al. 2002; Paternoster and Bushway 2009; Nugent and Schinkel 2016) with most research suggesting *“that the desisting identity must be a replacement self which is fundamentally different from the previous ‘offending self*” King (2013: 153). In addition, a study by Bubolz and Lee (2021: 846) found that role residual, or *“the lingering aspects of one’s former gang identity*” proved to be a relevant aspect in gang desistance but, in general, the literature on gang desistance and disengagement shows that the role of identity is an area of both empirical and theoretical paucity (Decker et al. 2014; Bubolz and Lee 2021). Therefore, in addition to documenting Jermaine’s experiences with non-offending, I will also pay attention to the role of ‘spoiled identity’ (Goffman 1963) in Jermaine’s process of desistance.

In this paper, I will use Jermaine’s story to illustrate that desistance emerges as a prolonged and fluctuating process, marked by intermittent periods of non-offending and occasional setbacks. Overall, it is a demanding journey that calls for persistence, emotional resilience, and sustained personal commitment from those seeking to exit street life. At the same time, desistance is marked by considerable ambiguity and is deeply embedded within specific social and structural contexts that significantly shape its trajectory. This paper proceeds as follows. First, I briefly sketch the contours of the Dutch gangland and the origin and development of the Dutch Crips gang as a contextual background to situate and understand Jermaine’s process of desistance. Second, I outline Jermaine’s story, in order to highlight key issues relating to both his act- and identity-desistance. The final section concludes with some broader reflections on the ambiguities of desistance and how these can condition post-gang trajectories.

## Dutch Ganglands

In the early 1990s in the Netherlands, there were concerns about youth who referred to themselves as ‘gangsta’, and who claimed to be part of gangs that were labelled either “Crips” or “Bloods”. The Hague—the third largest city in the Netherlands—was especially identified as the home of several Crips gangs. In 1994, local police in the city estimated that approximately 250 youths were active members in fifteen different gangs (Roks and Densley 2020). However, unlike the transnational diffusion of various Latin American gangs such as the Latin Kings or the Ñetas from the Americas to Europe (Brotherton 2007), the dissemination of Crips and Bloods gang culture and symbolism was the result of global flows of gang and street styles, a process that Van Hellemont and Densley (2019) have described as “*gang glocalization*”. In the words of Hazen and Rodgers (2014: 2), *“more mimicry than migration”. Given the stereotypical associations with the well-known American incarnations of the Crips and Bloods, the word* ‘gang’ was generally avoided in official accounts and scientific publications in the Netherlands or used ‘only with reluctance’ (Van Gemert et al., 2008: 8), although the media tended not to suffer such compulsions.

The case of one of these Dutch Crips ‘gangs’ that originated in the city of The Hague is well-documented. Part of their history is covered in articles in popular magazines, a book written by a journalist, in different shows on the Dutch national television, and a 90 min documentary. The book ‘Crips.nl’ (Van Stapele 2003) sparked my interest in this Dutch gang and resulted in ethnographic fieldwork for my master thesis in 2007 and for my PhD research between 2011 and 2014 (Roks 2018; 2019). The “h200d”, as my research participants called the neighborhood, was my main field site and the home base of the Dutch Crips since the late 1980s. Colloquially referred to as The Forgotten Village by residents, the “h200d” is located in one of the smallest neighborhoods in The Hague, home to about 1400 residents. The neighborhood can be characterized as an impoverished and highly racialized area (Roks 2019), with most residents of non-Dutch origin, mostly of Surinamese, Moroccan, Antillean, or Aruban descent (Roks 2019; Roks and Densley 2020).

Historically, the Dutch Crips in the “h200d” were a group of brothers, relatives, and neighborhood friends. Over the years, however, the composition of the gang changed. Friends of friends joined the set, and prison became a place where new members were recruited. This resulted in Crips that were born and bred in the local neighborhood, but also members that hailed from other parts of the city, and in some cases even from different cities (Roks 2018). During my fieldwork, the gang consisted predominantly of men with a Surinamese background, ranging in age from fifteen to forty years old. In general, they were involved in serious and violent criminality, including drug dealing (marijuana and powder cocaine) as well as the large-scale cultivation of cannabis. Several members were incarcerated over the course of my research, due to their involvement in stabbings, an assault on two police officers, possession of illegal narcotics, and weapon charges. Some research participants, however, combined these criminal activities with (often low paying) jobs, education, while some also collected unemployment benefits to make ends meet. Gang membership was moreover often ephemeral. At the start of my research in 2011, the h200d Dutch Crips consisted of approximately 50 members. Towards the end of my fieldwork in 2014, however, the number of active gang members in the set decreased, leaving only a core of 30 members, most of whom had been part of the Crips since the early 1990s. Jermaine was one of the 20 members that left the gang between 2012 and 2013 (Roks 2018).

## Jermaine’s Process of Desistance

In the remainder of this article, Jermaine’s process of desistance will be described chronologically. First, however, it is relevant to know how his criminal career started.

### From Temp Worker on the Streets to a Respected Member of the Dutch Crips

Jermaine grew up in the 1970s and 1980s with his mother and sister in a large city in the Netherlands, after migrating at a young age from Suriname, one of the former Dutch colonies. When I recently asked him to give a brief sketch of his childhood, he wrote:

I don't remember my mother hugging me or telling me she loved me. My mother must have done that. However, I only remember the harsh Surinamese upbringing. My mother meant well, but she didn't know any better. (…) Due to my harsh Surinamese upbringing, I hated my mother intensely. (October 30, 2023, email from Jermaine)

After not speaking to her for 16 years, nowadays Jermaine has a good relationship with his mother.

During a conversation in 2013, I asked Jermaine about the first time he committed a crime. After taking some time to answer, repeating that he had to dig deep into his memory, he stated: *“Not until I was in my early twenties, I think”*. Before that, Jermaine attended school and obtained a diploma as an auto mechanic. After his eighteenth birthday, he entered the military for a year then worked as a bouncer in Rotterdam for a time. In his early twenties, he started earning some money on the street, describing it as follows: *“It was simple, I was too lazy to work. I was looking for money. Why make it hard when there is an easy route?”* (Van Stapele (2003: 120). Van Stapele characterized Jermaine at this time as a street offending temp worker, not a person professionally engaged in a life of crime, but rather someone who did odd criminal jobs in the street to make some cash.

Around 1992, when Jermaine was in his early twenties, he decided: *“Fuck it, I’m not going to work”* (Van Stapele 2003: 121) and bought a shotgun, amongst other things to start robbing banks. After two years of more serious criminality, Jermaine got involved in a fight that escalated and ended up shooting a man in the leg who died due to an arterial hemorrhage. Jermaine initially faced spending twelve years in prison for manslaughter, but he was eventually sentenced to eight years, serving five and a half years incarcerated. This was the first time that Jermaine saw the inside of a prison, which turned out to be a turning point in his life. In jail, Jermaine met Raymond, the Dutch leader and founder of the Crips. In a personal note written in preparation for a guest lecture about his life at a school in Rotterdam, Jermaine looked back on this moment:

My tour of duty started in 1994. I got locked up because of an argument that really got out of hand. And during that sentence, I met Raymond. (…) We clicked from the start. He told me about the Crips. A group of guys who stand up for each other, who are down for each other. A big family, which appealed to me because I always wanted to have brothers. I've always had to fight alone. I had no one to back me up. (…) So, when Raymond told me about the Crips, I knew what I was going to be: a Crip. (November 27, 2013, personal document Jermaine)

After serving his prison sentence, Jermaine joined the Crips in The Hague, although he continued to live in the Rotterdam area. There, as he told me back in August 2013, he found what he was looking for: *“I had brothers now and we were tight. With them as a back-up I could take on the whole world. I finally got the love and appreciation that I always wanted.”*

### Sin’s Death

After being released from prison, Jermaine led a somewhat nomadic life, changing addresses and sometimes cities, mostly due to his frequent run-ins with the law. His romantic relationships were short-lived and he became a father a number of times, although his active street life interfered with seeing his children on a regular basis. Jermaine’s process of desistance was set in motion in 2005, when he sought assistance from a national prisoner aftercare organization to turn his life around. From a desistance perspective, Jermaine’s seemed to have a *“hook for change”* (Giordano et al. 2002), or a cognitive change that made him open to changing his life, motivated by a desire to be present in his children's lives. However, his efforts to find formal employment were unsuccessful, which he mainly attributed to his age, visible tattoos, criminal record, and skin color. In 2006, his daughter suffered a tragic accident cutting herself with a kitchen knife trying to make a hole in a belt. As the blade slipped, it hit an artery, leading to a very serious injury which put her in hospital where she remained in a vegetative state. This extraordinary sequence of events was a serious setback for Jermaine steering him back to the street and criminality. Exactly one year after his daughter's accident, Jermaine was stopped during a traffic control with police officers finding handcuffs, tie-wraps, and a firearm in his car. As a result, Jermaine ended up in prison again, this time for 18 months.

On being re-released from jail in 2009, Jermaine immediately picked up his old street life again. When I started my PhD fieldwork in 2011, Jermaine was not a daily presence in the h200d. He would, however, drop by a few times a month to talk to the Crips gang leader Raymond about *“projects*”—serious forms of crime about which neither Jermaine nor Raymond ever disclosed any specifics to me. In 2012, Jermaine and I became closer, partly because whenever we met up in the h200d neighborhood, he started offering to drive me back to Rotterdam where we both lived. During these thirty-minute car rides, I got to know Jermaine better. As we talked, I noticed that he often mentioned that he wanted to do something different with his life, and that he had ambitions to become a bodyguard. The first times he told me about these aspirations, I found it hard to reconcile the image of Jermaine as a bodyguard with his ongoing *“projects”* and his contacts with Dutch organized crime figures.

On the night of August 20, 2012, something happened that would reignite Jermaine’s process of desistance: his close friend and fellow gang member Quincy ‘Sin’ Soetosenojo was shot at close range in his hometown of Amsterdam and died from his injuries. Sin was someone Jermaine cared deeply about having lived together for some time and developing a special bond over the years. At the funeral, Jermaine was one of the people who carried Sin’s coffin to his final resting place. Sin’s death hit Jermaine hard. As we sat in the car talking in early September 2012, Jermaine confided that it was *“a very cold shower* and that he was still very emotional and cried a lot, especially when alone in his car. He also shared that he was not sleeping well and that he often woke up in the middle of the night and kept thinking about Sin’s death.

Sin’s death was also a defining moment of my PhD fieldwork. For many younger research participants, the passing of Sin initiated their gang disengagement (Roks 2018). Jermaine similarly started to gradually distance himself from the gang after Sin’s death. He had initially only told me about his future aspirations for legal work, but some weeks after Sin’s death, he started to openly discuss his ideas of becoming a bodyguard and no longer relying on the street economy with people around him. Based on their laughter and jokes, most older gang members did not seem to take Jermaine’s ambitions very seriously. Initially, I had my doubts too, given his involvement in various *“projects”* on the street. During yet another car trip to Rotterdam, I briefly mentioned that I was there for him if he needed help realizing his legal ambitions. At first, this was just something I said out of courtesy, and, moreover, something I also said to at least five other gang members around the same time. None of them took me up on my offer, but Jermaine did.

From December 2012, Jermaine visited my office at the university every week to take steps to leave the street and gang life behind. Despite having offered to help him, I had little idea how to help someone who wants to turn their life around. Soon, however, I noticed that the help Jermaine required was mostly practical in nature. He wanted to start collecting unemployment benefits, find a house and a job, and to do so he had to fill in various forms and submit documents to different authorities. Jermaine asked me to read and correct his filled-out forms and other letters, and to print whatever relevant documents he needed.

Although Jermaine was seriously interested in becoming a bodyguard and actively looked for a place to start his training, he admitted that he was still *“doing his thing on the street”.* Jermaine however quickly realized that if he wanted to work in this industry, he would have to submit a so-called VOG—a statement of (good) conduct. This meant that he could not have any outstanding prison sentences, fines, or debts and had to make a clean slate leading him to report to the police to serve a remaining sentence of 88 days in prison from a prior conviction in 2007. During this period, we kept in touch daily via WhatsApp. One day he did not respond to a question and when I saw on his WhatsApp profile that he had not been online for a while, I concluded that he either turned himself in or was arrested. Gang leader Raymond confirmed my suspicions that he was back in jail after having been arrested during a raid on his mother’s house in January 2013.

### Playing the “Game”

In May 2013, Jermaine was released from prison and picked up where he had left off before he went to jail. He restarted his application to receive unemployment benefits, his search for a home, drew up a resumé and applied for various jobs. Our conversations highlighted how Jermaine’s accumulation of street capital (Sandberg 2008) conflicted with the norms and expectations of mainstream society, complicating his process of desistance. For example, he had to adopt unfamiliar roles when interacting with authorities—roles that differed significantly from those he was accustomed to in street contexts. Jermaine’s frustration triggered behavioral responses shaped by his street socialization, as the following message on July 9, 2013, illustrates well:

IF IT REALLY REALLY DOESN'T WORK THEN I'M GOING TO DO MY THING AGAIN ON THE STREET.

During this period, the motivation to really change his life seemed to be the deciding factor:

Hahaha yes...I'm over here smiling: Jermaine 187 NHC is writing his resume...hahaha I'm so fed with this...but I'm getting my own house no matter what… so they want me to apply to jobs, all good… let tha game begin... what i told you: chess. (...) I have to play this game so that I can get my benefits and a house... you MUST show that you are actively looking for work... and only then will they process your application... no matter what... I will get my own osso [house] ...that's my goal... (July 11, 2013, conversation with Jermaine via Whatsapp)

In our discussions during that period, Jermaine described his approach to navigating unfamiliar situations, including meeting requirements and expectations of Social Services, as *“playing the game”*.^3^ Referring to this as a game signaled the performative nature of Jermaine’s endeavors at that time. On one level, he gave the impression that he was motivated to find a job to reach certain goals, like collect unemployment benefits or be allocated a home. On the other hand, he constantly made references to his gang identity with his tattoos of '187' under his chin (a reference to the police code for murder in some US states) and ‘NHC’, an abbreviation of Neighborhood Crip, all part of his symbolic “appearance*”* (Goffman 1959: 34) as a Crip. As Jermaine transitioned away from street life, markers of his gang identity seemed to become more salient within the context of his desistance process.

After writing up his resumé, Jermaine sent it to me for editing. In the email, he stated that he initially though that there would be a gap of twelve years since his last regular job as a bouncer. However, in the process of putting his resumé together, he realised that his last ‘normal’ job went back to 1994, almost 20 years previously. In the document, Jermaine put *“WANTS TO WORK*” in all caps above his resume. Lacking what Bourdieu (1985) describes as cultural capital—specifically, a limited level of education and a lack of experience in the formal job market—Jermaine’s job applications were unsuccessful. Eventually, however, Jermaine was accepted in a work program organized by the Dutch Social Services, and at the end of August 2013, started a job in in horticulture. As he wrote to me after a few days:

The workplace is Fucking biiiiiiiiiiiig... and the people are chill... just got dropped off and now I'm going to cycle home... you know what's funny... I'm wearing the work clothes that I use for plants [weed]... now I'm working with plants again but in a different way... when I'm cutting the plants I am laughing out loud... I'm going to cycle home, app you later. (August 27, 2013, conversation with Jermaine via Whatsapp)

Jermaine’s linking of his new pro-social activities to his past criminal experiences with weed cultivation provides a clear illustration of accumulated street cultural capital (Sandberg 2008). Given his life trajectory, it is not surprising that his frame of reference when it comes to work is crime, something that he has been engaged in for most of his adult life, including the approximately nine years he spent in jail. This did not stop him from doing well in his horticulture job, but he had to leave early several times due to problems with the Social Services administration. For example, information was missing in his application for employment benefits, and as a result, he was unable to complete the work program, rendering him ineligible for a long-term employment contract. Nevertheless, Jermaine left such a positive impression on his boss, that the latter wrote that: *“If Mr. Jermaine has his affairs in order (financially, housing, *etc*.), as well as peace of mind, he is welcome to report to us again and we will be happy to see what we can do. With his enthusiastic attitude he was an example for others”.*

### A Battle Between the Crip Angel and the Crip Devil

After the work program, Jermaine conjured up the idea of training to become a painter at a school in Rotterdam, specifically to enhance his limited job qualifications. During the intake for the training program, Jermaine talked at length about his criminal past. Based on this conversation, he was invited to give a couple of talks to at risk youth at the school. Fully motivated, Jermaine started working on a PowerPoint presentation detailing his life experiences, putting together an extensive script with edited videos and images and asked me for feedback. After a few such presentations, he forwarded the following email to his family and friends:

Do you know how one of the teachers addressed me: “Hey Jermaine can I ask you something?”... Now Jermaine is not only associated with the street but Jermaine is also someone who wants to show youth that they should stay off that fucking street. EVERYTHING HAPPENS IN YOUR LIFE FOR A REASON. (December 19, 2013, email from Jermaine)

A great deal of pride was expressed by Jermaine’s friends and family in reaction to his e-mail, which pleased him. In concordance with Maruna (2001: 87) redemption script, Jermaine drew a lot of energy from interacting with young people and the idea of being able to play a meaningful role in their lives based on, as Jermaine put it, his *“mistakes”,* appealed to him enormously. Additionally, he felt appreciated. Taken together, it seemed giving these presentations to at risk youths contributed significantly to keeping Jermaine off the streets.

At the same time, however, I got a glimpse of how Jermaine’s street capital was hindering his process of desistance. For instance, Jermaine was regularly frustrated with bureaucratic procedures, and he often had the feeling that he was being treated unfairly. Especially in the process of collecting unemployment benefits: Jermaine felt that he had to wait a long time before his application was approved and he received his money. Additionally, he did not receive the total amount he expected. When I asked him why this was the case, he said that Social Services had stated that he had not fulfilled all the mandatory tasks and assignments. Jermaine had kept a remarkable amount of documentation about the latter, but he was still unable to provide the correct information, much to his frustration. During this period, he mentioned that he *"really has to unlearn certain things",* that *“his hands are itching”*, that he was struggling to keep it together and not resort to violence. On several occasions, I had to calm him down, either via messages on WhatsApp or on the phone.

Towards the end of 2013, Jermaine’s application to collect unemployment benefits was approved. Moreover, he had been assigned housing that he was slowly furnishing with the help of family and friends. Jermaine was busy with his bodyguard training and spent a lot of time in a gym. Additionally, he frequently gave presentations at schools in Rotterdam about his experiences on the streets. He made some money from this, but not enough to make ends meet. Unlike a number of my other research participants, Jermaine led a fairly frugal life and had little interest in expensive clothing or an extravagant lifestyle. During this period, I occasionally supported Jermaine by giving him money for groceries, buying him a movie subscription, or lending him a bicycle. He generally accepted my help with some reluctance and repeatedly indicated that he had to put aside his pride and his manhood to do so. Yet, at that moment he had no other options, and he would also ask other people close to him for small financial contributions.

Even though Jermaine was doing well, I noticed he was struggling not to get drawn back into his old street and gang life. In the aforementioned personal document, he described this graphically as a *“battle between the CRIP ANGEL and CRIP DEVIL: the angel who keeps me from going back to the street game and the devil who keeps telling me to go back to my old life”.* For Jermaine, the Crip Angel encompassed the good steps Jermaine had taken, such as the improving the relationship with his children, getting his own house, and receiving unemployment benefits. The Crip Devil, however, epitomizes Jermaine’s negative connotations tied to his psycho-social embeddedness in street and gang life. At that time, finding a balancing between his Crip Angel and Crip Devil appeared to be an extremely precarious endeavor for Jermaine.

At the beginning of 2014, I hadn't spoken to Jermaine for a few weeks, and he told me that he had been back to the h200d neighborhood in The Hague to meet with the Crips gang leader Raymond. He described having done so as *“a protection project”*, and emphasized that it was an extension of his training as a bodyguard and not that risky. This highlighted something that also complicated Jermaine's process of desistance: his social network was comprised of few people who could help him find a regular job, but he regularly received offers to make money on the streets. This surplus of street social capital (Ilan 2013) led to a significant moral conflict, as he highlighted during a WhatsApp conversation on March 10, 2014:

Roks... this... is... not... funny... any more... it's like the devil is playing with me.... [But] I can really make a lot of duku [money].... I saw a mati [friend], VERY INTERESTING but still no… [But] when I was busy on the street things like this didn't come... I do have a serious inner conflict right now: Crip Devil vs Crip Angel… But then I think about my beautiful moments with my daughter… MONEY IS NOT EVERYTHING BUT EVERYTHING IS EVERYTHING ABOUT MONEY... I am tired of depending on others... but that's the old Jermaine talking again… (March 10, 2014, conversation with Jermaine via WhatsApp)

### “I am No Longer Active, but I will Always Be a Crip”

Slowly, Jermaine started building a social network that provided him with non-criminal opportunities. In May 2014, he landed a job as a youth coach through someone he met while giving presentations in Rotterdam about his gang life. He proudly shared photos of himself signing the first employment contract of his life. At that point in time, Jermaine’s desistance trajectory showed intermittent setbacks; nonetheless, securing employment signaled a shift toward sustained non-offending. Notably, in the preparatory document previously cited, Jermaine underscores the centrality of identity in his process of desistance:

Acquaintances and friends I haven't seen for a long time always ask me about The Hague, whether I'm still with them or whether I'm still going there. I explain to them that I haven't seen them in a long time and I'm in the process of finding work, that I'm high on the list for my own home. Then, they are happy to hear that I have taken a different turn in my life and they say: you are no longer in The Hague BUT YOU STILL WEAR BLUE! You know what it is, I'll always wear blue, I'll always be blue. I am no longer active, but I will always be a Crip. I made a conscious choice about that in '94. It says on my body: TRU BLU TILL MY CASKET DROPS. CRIPING has cost me too much. It cost my freedom, it cost my relationship with my partners. AND WORST OF ALL IT COST MY RELATIONSHIP WITH MY KIDS. So yes, I will always wear blue… [BUT] THAT SHIT COST ME TOO MUCH.” (November 27, 2013, personal document Jermaine)

This excerpt highlights the importance of the Crip identity for Jermaine, something that is deeply rooted and has come at a price. Although Jermaine described that he still wears blue and is still a Crip, something changed in this respect in June 2014, when he gave a guest lecture on his life in a BA course on “Criminal Careers” that I teach at Erasmus University Rotterdam. Jermaine introduced himself by his gang name, followed by *“AKA Jermaine, member of Rollin 200 Main Triad Neighborhood Crips*”. He told the students that he had been an “*active*” gang member for many years, but that he was no longer “*gang banging*” and had found a new meaning and interpretation of what it meant to be a Crip. It was not only his introduction that was symbolic in relation to his gang identity desistance that day, but also the way he was dressed. He wore a red tracksuit during the lecture, a color usually associated with the Bloods, the Crips’ sworn enemies. The tracksuit was a deliberate choice, as became evident by the big smile on Jermaine’s face when a student asked him why was wearing the color red as a Crip. “*A very good question”*, answered Jermaine, before going on to explain that his mother had also been shocked when she saw him in a red tracksuit, but that it represented a new phase in his life, because wearing ‘red’ did not make him less of a Crip. This interaction illustrates that Jermaine’s desisting identity was not, as King (2013: 153) contends, *“fundamentally different from the previous ‘offending self’”,* and indeed incorporates *“certain characteristics or behaviors previously associated with the ‘offending self’”.* Crucially, Jermaine’s continued affiliation with the Crip identity did not appear to undermine his process of desistance.

After my PhD fieldwork, Jermaine and I kept in touch. On average we spoke a few times a month. Often, he would reference his old way of life, stressing how happy he was about his current endeavors. When I re-interviewed him in May 2023, Jermaine reflected on how much his life had changed the last decade, particularly compared to his peers. Just eighteen months earlier, in July 2021, some of his “homies” received long prison sentences in a high-profile case that centered on several contract killings in the Dutch criminal underworld. Others, though, had managed to turn their street and gang lives around and were now leading rather conventional lives, including working regular jobs. Like Jermaine, the broader socio-structural context of the Netherlands also facilitated their processes of gang desistance: they did not suffer from residual consequences after gang disengagement such as still being treated like a gang member by the police or criminal justice system, being included in gang databases, or confronted with former gang members or rivals in daily life (Roks 2018).

During the interview in 2023, Jermaine’s emphasized how his experiences on the labor market was challenging, specifically because he had had to change jobs several times over the past decade. One time, this was due to budget cuts. Another time his contract did not get renewed, and he was forced to leave. However, Jermaine kept his head up and was motivated to find new opportunities. After being a youth coach, he worked as an absenteeism coach at a school. Some years later, he started working as a youth worker in Rotterdam. In all these jobs, Jermaine has worked with street-oriented youth, individuals who remind him of how he used to be. The common point of all these different occupations is that Jermaine did not immediately have to renounce his identity as a Crip. Indeed, it was his Crip past and his old identity that provided him with an edge in relation to these his new jobs, and imbued his new identity with a certain degree of legitimacy, as it made him valued both by the “at-risk” youth he interacted with, as well as many of his teacher and social worker peers. Rather than serving as a hindrance, his criminal and gang-related history functioned as a positive asset in his process of gang desistance.

## Conclusion

This paper examined Jermaine’s process of desistance by ways of longitudinal ethnographic research. Owing to this methodological and physical proximity, the study adopted a phenomenological perspective to explore how Jermaine, as a member of a Dutch street gang earning money through illicit means, attempts to redirect his life trajectory. His life story aligns with the work of Maruna (2001) and Rodgers (2023), suggesting that desistance cannot be understood as a clearly demarcated moment in time. Rather, it emerges as a long and dynamic process, marked by alternating periods of offending and relapses, and shaped by what Nugent and Schinkel (2016) describe as the ‘pains of desistance’—experiences that help explain why desistance, in the words of Bottoms and Shapland (2011), can be seen as a ‘bumpy road’. Jermaine’s story, in particular, illustrates that this process demands effort, persistence, resilience, and willpower on the part of the desister.

In Jermaine’s story, two interrelated spheres of desistance were highlighted. First, *act-desistance,* or the process of no longer engaging in criminality (Nugent and Schinkel 2016). From 2012 onwards, Jermaine successfully collected unemployment benefits, applied for several jobs, and found a house. Since his adolescence, Jermaine’s socialization had taken place on the streets, resulting in the development of criminal skills and a street-oriented network. This was evident in his labour market experiences: he had little in the way of relevant work experience for formal jobs, a substantial criminal record, a tendency to resolve problems by resorting to violence, and a social network that mainly provided him with criminal opportunities. Jermaine's story, therefore, showcases how cultural and social street capital interact and can complicate a transition to the formal economy of labour (cf. Sandberg 2008; Sandberg and Pedersen 2011; Ilan 2013).

Secondly, Jermaine's story underscores the importance of identity in the process of desistance. However, unlike what Giordano et al. (2002) and Paternoster and Bushway (2009) argue, Jermaine's story illustrates that *identity-desistance* does not always involve a fundamental and intentional shift in self-image. Consistent with the work of Maruna (2001), Rodgers (2021), and King (2013), Jermaine’s story demonstrates that desistance does not entail a complete rupture from his street or gang identity, but rather a redefinition that retains and reconfigures aspects of the former self. This constitutes an important insight within the literature on (gang) identity desistance. At a fundamental level, Jermaine’s story challenges the normative—and arguably false—binary between a pro-social and criminal identity that underpins much of the existing research on (gang) identity desistance. Specifically, it raises two significant questions regarding identity within the desistance process.

First, it prompts the critical question of whether the adoption of a new, conventional identity is even feasible for street-oriented, gang-involved individuals. For instance, Contreras’ (2013: 235) account of young men involved in the drug economy in the Bronx, New York, uncovered how their street cultural capital impeded their entry into the legal workforce, but also how *“after failing as drug robbers, they self-destructed as they fought against their new identity as fallen stars”.* Similarly, the work of Lee and Bubolz (2020) attunes us to the importance of residual stigma as a consequence of gang pasts in terms of (anticipated) stigma from police and society at large. In that light, Jermaine’s story raises the fundamental question of whether transforming or substituting one’s ‘spoiled identity’ (Goffman, 1963) is a realistic undertaking, given the lived experiences of desisters. This shows, as Maruna (2025: 13–14) argues, that desistance is not merely about individual process of change, but also necessitates a societal change—requiring shifts in how we perceive and engage with street-oriented, gang-affiliated, or system-impacted individuals.

Building on this, Jermaine’s story illustrates that the construction of a new, pro-social identity did not require the complete erasure of his former self. Indeed, the concluding segments of his story reveal how he retains certain elements of his gang identity and integrates them into his emerging role as a youth coach, absenteeism mentor, or youth worker. His criminal and gang past became a positive rather than a negative resource. In Jermaine's case, his age, criminal career, gang history, and new jobs all come together to play an important role in explaining why he has been able to hold on to parts of his old identity. Like Jermaine, more desisters who have distanced themselves from their old life, but who still rely on parts of their criminal or gang past in new roles and identities (De Jong 2016; Brotherton 2025; Maruna 2025). Precisely because they, each in their own way, can still draw on part of their old criminal or gang selves in their new, more conventional identities, they are able to refrain from relapsing into old patterns of behavior. Therefore, people like Jermaine are well-suited to guide, support, and inspire others in their processes of desistance (Maruna 2001: 117–130; Maruna 2025: 13–16).

## Footnotes

1 Jermaine is a fictitious name.

2 As part of the sub-project “Gangsters” of the European Research Council (ERC)-funded project “Gangs, Gangsters, and Ganglands: Towards a Global Comparative Ethnography” (grant no. 787935), I re-interviewed Jermaine in May 2023. Specifically, we discussed his process of desistance, following the chronology presented in this article. Moreover, in the process of writing this paper I contacted Jermaine a few times to ask specific questions about certain events in his life. After reading a draft version, Jermaine gave his written informed consent regarding the use of all the data—consisting of observations, informal conversations, WhatsApp conversations and e-mails—that I have collected over the years.

3 In his research in Sierra Leone, Utas (2014) also came across research participants using this expression. *“Playing the game”,* Utas (2014: 175) writes, could be seen “*as a kind of social navigation fitting neatly with Bourdieu’s (2000) notion of illusio, routinized forms of social interaction where individuals are aware of their bets”.*
